# Ozonolysis of Lignin: From Extensive Degradation to Selective Ring‐Opening Oxidation

**DOI:** 10.1002/cssc.202501853

**Published:** 2026-02-12

**Authors:** Alexandros E. Alexakis, Mika H. Sipponen

**Affiliations:** ^1^ Department of Chemistry Stockholm University Stockholm Sweden

**Keywords:** biomass, materials, pulp, selectivity, sustainable

## Abstract

Despite its potential as a renewable feedstock, lignin, a major component of plant cell walls and a by‐product of the pulp and paper industry, has long been underutilized. Historically, pulp mills employed ozonolysis, a powerful oxidative process, to delignify cellulosic fibers and break down lignin. The ability of this technique to modify lignin structures has attracted attention in recent years, creating opportunities for the development of novel lignin‐based materials with improved functions. This review revisits the use of ozone as a bleaching agent and bridges its historical role in pulp processing with its emerging potential as a selective oxidative tool for lignin modification in materials chemistry. We discuss the fundamental chemical processes, significant developments in ozonolysis technology, and its potential to functionalize sustainable biomaterials. We also give our perspectives on the present obstacles and potential opportunities for ozonolysis optimization in lignin valorization.

## Graphical abstract

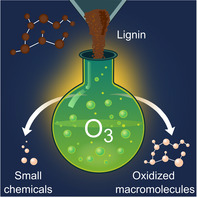

## 1 Introduction

**FIGURE 1:**
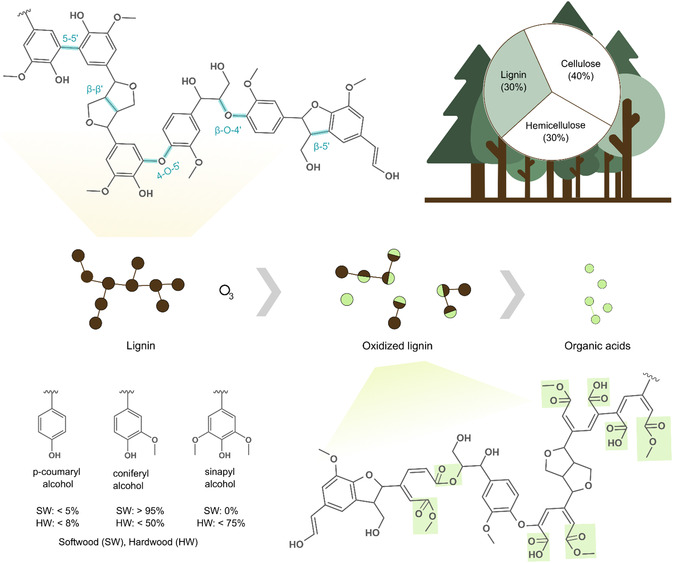
A schematic representation of the composition of wood, with focus on the lignin component. The three monolignols and key linkages in a possible molecular structure of lignin are shown alongside a schematic of ozonolysis of lignin. The first product is a partially oxidized, fragmented lignin that then yields small organic acids under extensive ozonolysis.

The ubiquitous presence of lignin in the woody tissues of all terrestrial plants, constituting ≈20%–30% of the dry weight [1], makes lignin one of the largest reservoirs of organic carbon on Earth. In contrast to the relatively homogeneous polysaccharides cellulose and hemicellulose, lignin is a complex, branched phenolic macromolecule composed of one or more of the three primary monolignols: p‐coumaryl alcohol, coniferyl alcohol, and sinapyl alcohol (Figure 1). Various ether and carbon–carbon bonds bind these units together to form a heterogeneous macromolecule that varies in terms of molecular weight distribution, interunit bonding pattern, and concentration of functional groups based on plant species and extraction methods [2]. This heterogeneity becomes apparent when comparing some of the most common technical lignins, namely kraft lignin, organosolv lignin, alkali lignin, and lignosulfonates, with that of native lignin present in plant tissues.

In nature, lignin is formed through the oxidative polymerization of monolignols, resulting in a complex, recalcitrant aromatic biopolymer that imparts mechanical strength and hydrophobicity to plant cell walls. Paradoxically, the same structural stability that is vital to plant integrity has long posed a challenge in the industrial processing of biomass. To access cellulose fibers, lignin is removed during pulping and has historically been regarded as an industrial by‐product with limited commercial applications, mainly restricted to its use as a low‐value fuel for energy recovery in pulp mills and biorefineries. However, the increasing interest in sustainable materials and bio‐based alternatives has spurred research into its valorization [3]. To fully utilize lignin in high‐value applications or to convert it to bio‐based chemicals, adhesives, composites, and nanomaterials, effective processing and modification methods are required.

Nature's oxidative construction of lignin through radical coupling is being mirrored in reverse by industrial strategies aimed at its depolymerization. In the production of cellulosic fibers, delignification typically involves harsh conditions that result in condensed and oxidized lignin residues, complicating subsequent valorization. To enhance pulp purity and brightness, oxidative bleaching stages are introduced. Ozone (O_3_) has historically played a key role in pulp bleaching. The match between electrophilic ozone and the chromophoric, aromatic structures generated during pulping enables targeted disruption of conjugated double bonds and aromatic rings. This not only deconstructs color‐causing moieties but also fragments the lignin backbone, offering a selective and potentially tunable route to lignin modification.

Ozonolysis has been widely used in the pulp and paper industry for lignin removal during bleaching, offering an environmentally friendly alternative to chlorine‐based treatments [4]. In contrast to bleaching, where the aim is to degrade the chromophoric lignin units and achieve pulp fibers at high brightness and high yield, less intensive ozonolysis holds potential for selective modification of lignin. Such reactivity finds new relevance as a platform for developing controlled oxidation strategies in the context of lignin transformation [5]. Recent advancements have demonstrated that ozonolysis can modify lignin at a molecular level, therefore enhancing its solubility, reactivity, and functional properties for advanced material applications [6, 7].

Previous reviews related to the oxidative degradation of lignin have focused on the high‐value chemicals obtained through oxidation processes involving enzymes and organometallic catalysts [8, 9]. In this review, we explore the evolution of ozonolysis from its conventional use in pulp mills to new technologies involving lignin‐based macromolecules and their intended use in materials chemistry. We will discuss novel applications of ozonated lignin materials while addressing key advances in process optimization and focusing on the fundamental chemistry of lignin ozonolysis. Furthermore, we will present the challenges associated with ozonolysis, including reaction selectivity, scalability of the process, and environmental considerations, as well as focus on future research directions to fully harness the potential of lignin valorization through ozonolysis.

## 2 Short Historical Perspective of Ozonolysis of Lignin

Ozonolysis was first introduced in the early 20th century as a means of bleaching in pulp mills. Before ozonolysis, the main technologies involved chlorine‐based bleaching methods, which produced chlorinated aromatics that are difficult to remove from the effluents. These compounds are environmentally persistent and bioaccumulative, raising significant health and environmental concerns. Particularly, many of these chlorinated aromatics are now recognized as part of the broader class of persistent organic pollutants (POPs) and share similarities with substances categorized as per‐ and polyfluoroalkyl substances (PFAS), especially in terms of their resistance to degradation and their long‐term environmental and health impacts [10, 11]. Ozonolysis emerged as an environmentally friendly alternative, which not only reduced the production of chlorinated by‐products but also decreased the chemical consumption and improved the overall pulp brightness [4, 5, 12, 13]. These advantages made ozonolysis a crucial part of the pulping process, and its integration into various stages contributed to more sustainable paper production [14].

In the 1980s and 1990s, significant advancements were made in optimizing ozone bleaching sequences, leading to the development of modern elemental chlorine‐free (ECF) and totally chlorine‐free (TCF) bleaching technologies [4, 12]. To date, there are more than 22 pulp mills worldwide that have implemented ozone as part of their multistage bleaching process [15, 16]. These pulp mills are located in Sweden, Finland, Germany, Slovakia, Austria, Portugal, Spain, Brazil, India, and Japan [15, 16]. Both the development and adoption of TCF and ECF bleaching were influenced by environmental policy and market forces [17]. As concerns grew about dioxins and adsorbable organic halogens (AOX) in mill effluents, regulatory pressure and public demand in Europe helped accelerate the phase‐out of elemental chlorine and increased the use of both ECF and TCF processes, with TCF finding its strongest niche in Scandinavia and Germany's eco‐conscious markets [18]. TCF‐bleached pulp reached significant regional penetration in Europe in the late 1990s, though globally it remained a smaller segment than ECF. In North America and much of South America, ECF bleaching predominated, driven by regulations that phased out elemental chlorine without requiring TCF, along with considerations of cost, yield, and existing infrastructure [19]. In Japan, pulp producers similarly transitioned toward ECF to reduce harmful discharges, with TCF achieving only limited use relative to ECF [20]. Thus, regional environmental policies and market demand shaped the relative uptake of TCF vs ECF, alongside technical and economic considerations. Recent research on ozonolysis is focused on the optimization of the whole process and minimizing the energy consumption [13]. Advances in reactor design, ozone dosage control, and hybrid bleaching systems have further increased the industrial feasibility of ozonolysis [21]. Although ozonolysis has become an integral part of the bleaching process due to its efficiency and environmental impact [21, 22], chlorine dioxide (ClO_2_) bleaching remains the dominant method globally. Additionally, the formation of adsorbable organic halogens, dioxins, and furans has been associated with ClO_2_ bleaching. This highlights ozone's potential for a more sustainable bleaching process [23, 24].

## 3 Mechanism of Ozonolysis in Lignin Degradation

### 3.1 Fundamental Chemistry of Reactions between Ozone and Lignin

**FIGURE 2:**
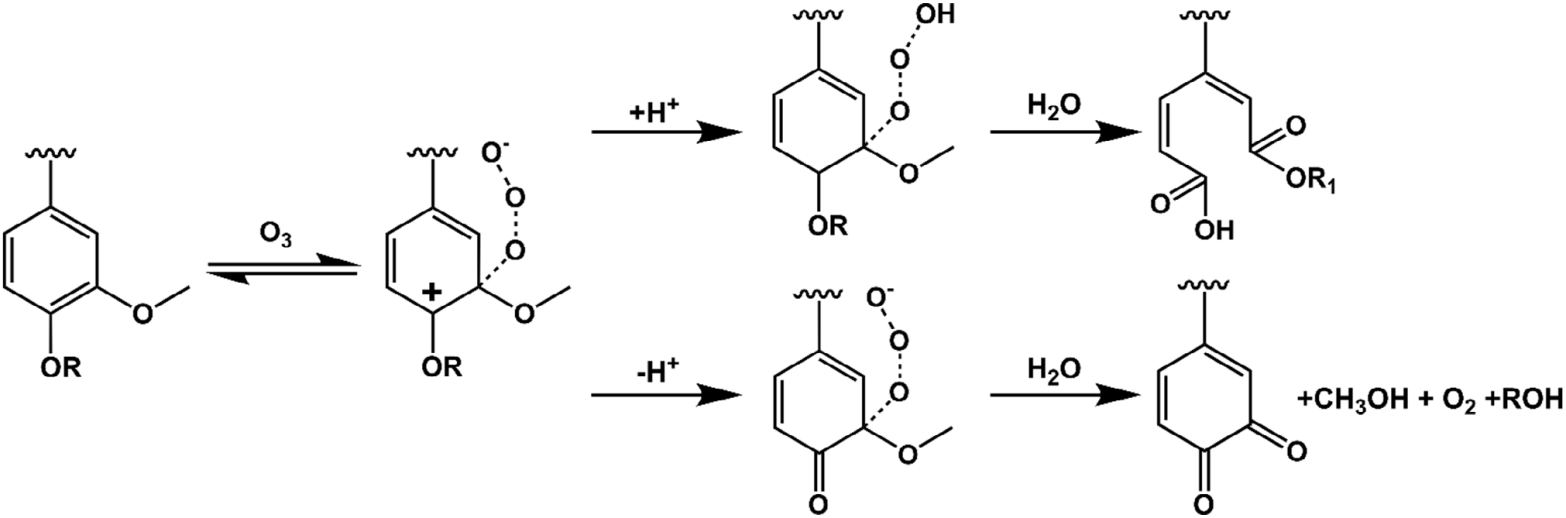
Chemical pathways showing the degradation of lignin with ozone, focusing on the attack of ozone on the aromatic ring of lignin [22]. In this context, “R” and “R1” can either be a hydrogen or a carbon linked to another lignin moiety. The reason for distinguishing between them is the chemical process they are involved in. The reaction routes strongly depend on pH and solvent environment, as will be discussed in the next section.

Ozone (O_3_) is a highly electrophilic oxidizing agent. It is commonly generated on‐site from oxygen (O_2_) either by applying an electrical field or UV light [25, 26]. In both cases, O_2_ molecules split into O atoms and quickly attach to unreacted O_2_, forming O_3_. The reactions of ozone with the various functional groups and interunit linkages in lignin (Figure 1) are complex and strongly influenced by multiple parameters, including pH and solvent choice. This is why the majority of the literature revolves around ozonolysis studies performed on model lignin compounds. Based on these studies, some generalizations can be drawn. Specifically, the rate of ozonation of lignin‐related aromatic moieties decreases in the following order: stilbenes > styrenics > phenolics > muconic acid intermediates [27, 28]. Additionally, the type of lignin, i.e., softwood versus hardwood, plays a key role in ozonation, as the number of methoxy groups significantly affects the rate of ozonolysis. The reactivity follows the trend: sinapyl > coniferyl > p‐coumaryl [29, 30]. The general consensus is that ozone reacts with the aromatic rings and double‐bonded side‐chains of lignin [31]. The reaction starts with ozone reacting with electron‐rich sites of the aromatic rings, most notably at the C3–C4 positions, resulting in unstable ozonides. These ozonides quickly decompose into muconic acid derivatives, aldehydes, and carboxylic acids, effectively breaking down lignin's structure (Figure 2). To highlight the complexity of the lignin structure and the resulting complex oxidized products, ozonolysis studies have been performed on model lignin dimers with common interunit linkages, namely *β*‐O‐4’, *β*‐*β*’, 5‐5’, and *β*‐5’, among others [27, 28, 31, 32, 33, 34, 35]. Special attention was given to the production of erythonic acid and threonic acid diastereomers, which are considered diagnostic products of the oxidative cleavage of *β*‐O‐4’, where the in‐between ratio reveals the original stereochemistry of the linkage. It must be noted that although these reactivity scales are useful, they need to be used with caution as they are based on model compounds, whereas the ozonation of lignin biopolymers might differ due to the many interconnected variables, which will be discussed in the next sections.

### 3.2 Reaction Pathways in Ozonolysis

When ozone reacts with the aromatic rings of lignin, it forms unstable ozonide intermediates, which, through rearrangement and cleavage, break down the aromatic ring into smaller fragments (Figure 2) [27, 36]. These fragments include dicarboxylic acid moieties, such as muconic acids, which contain two carboxylic acid (—COOH) groups at the chain ends or quinones that are quite unstable and readily rearrange to form acidic moieties.

The structure of lignin is composed of different interunit linkages. In native lignin, the most prevalent interunit bond is *β*‐O‐4’, having a crucial role in the three‐dimensional structure of lignin (Figure 1). During ozonolysis, these ether bonds are cleaved, resulting in a reduction of the molecular weight of lignin and, consequently, improving the processability and solubility of the products compared to the original macromolecules [37]. Asa result, low‐molecular‐weight aromatic compounds, such as vanillin, syringaldehyde, and 4‐hydroxybenzaldehyde, are produced in varying amounts depending on the ozonolysis parameters. Specifically, when expressed relative to the weight of lignin, the yield of vanillin ranges between 1 wt% and 8 wt% for hardwood and grass lignins, syringaldehyde is ≈12 wt% for hardwood, and 4‐hydroxybenzaldehyde ranges between 3 wt% and 5 wt% for grass and acetosolv lignin [38, 39, 40]. These low molecular weight aromatic chemicals released can be used in various applications, such as functional additives in biofuels and bioplastics [39, 41, 42].

Apart from the aromatic rings and ether linkages, lignins also contain oxygenated aliphatic side chains. In addition to the presence of aliphatic hydroxyl groups (—OH), the carbon atoms in the aliphatic chains participate in interunit linkages, which complicates the degradation chemistry during ozonolysis (Figure 1). In general, ozonation of these side groups introduces additional oxidized functional groups, making the lignin structure more polar [27].

### 3.3 Influence of Process Variables

**FIGURE 3:**
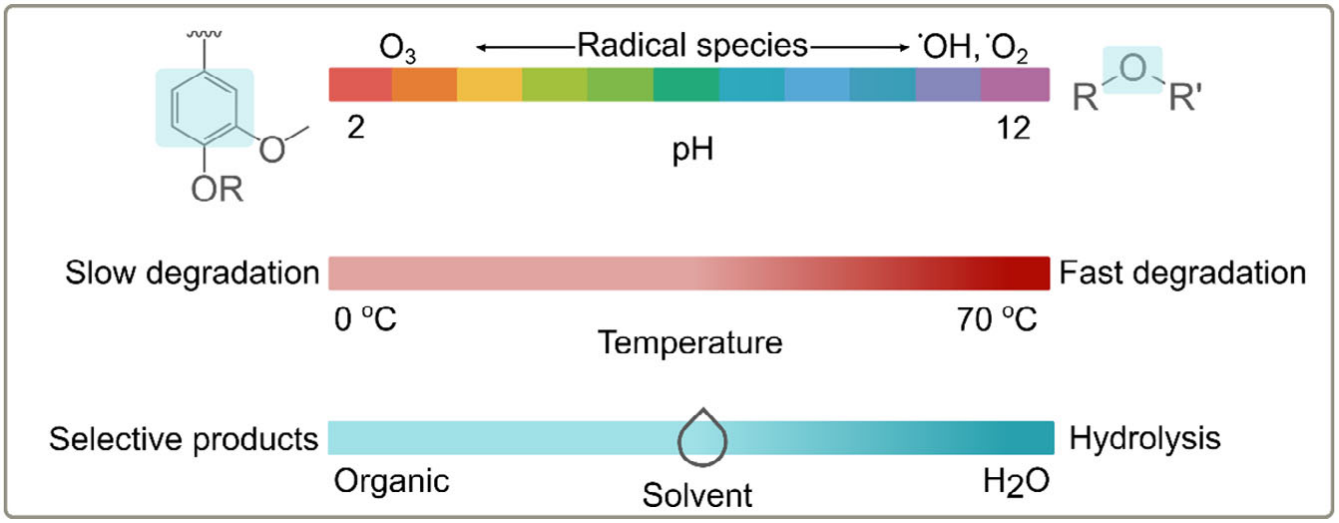
Schematic representation of the main parameters that influence the ozonolysis of lignin and how they can be tailored to target specific compounds.

Several parameters influence the efficiency of lignin ozonolysis. Below, only the main variables are discussed (Figure 3). Ozone solubility is a key parameter that determines the degradation pathway when reacted with lignin. Generally, ozone is poorly soluble in water. However, the optimum pH range that favors its solubility is between 2 and 6, which is also the operating window used in pulp bleaching [43]. Within this pH range, ozonolysis favors the oxidation of aromatic rings and side chains that contain double bonds. The slightly acidic environment causes the protonation of hydroxyl and carboxylic acid groups, which, as a result, increases the reactivity of certain bonds, thus promoting a more controlled degradation of lignin [7, 27]. Additionally, the elevated proton concentration in the reaction medium facilitates the formation of more stable ozonide intermediates, hence following the Criegee pathway [44]. At pH values below 2, ozone reacts with hydronium ion, leading to a reduced solubility [43, 45]. This is due to the formation of protonated ozone (HO_3_^+^) [43, 46].

On the other hand, at alkaline pH, ozone solubility decreases for other reasons. Contrary to acidic conditions, at alkaline pH, this reduction in solubility is related to the auto‐decomposition of ozone, accompanied by the formation of peroxide radicals (HO_2_^−^) [43, 47]. Further reaction of these radicals with ozone leads to the formation of multiple radical ions, which in extension act as initiators for the decomposition of ozone, as shown in the general scheme below:

$$O_{3}+{\mathrm{OH}}^{-}\;\to \;{\mathrm{HO}}_{2}^{-}+O_{2}$$

$$O_{3}+{\mathrm{HO}}_{2}^{-}\;\to \;\cdot O_{2}^{-}+\cdot \mathrm{OH}$$

$$O_{3}+\cdot O_{2}^{-}\;\to \;\cdot O_{3}^{-}+\cdot O_{2}$$

$$\cdot O_{3}^{-}+H_{2}O\;\;\to \;\cdot \mathrm{OH}+{\mathrm{OH}}^{-}+O_{2}$$

Under alkaline ozonolysis conditions, a variety of reactive radical species (e.g., hydroxyl and superoxide radicals) are generated, leading to a largely unselective oxidation process. These radicals promote not only the cleavage of carbon–carbon double bonds but also the accelerated oxidative scission of lignin's *β*‐O‐4’ ether linkages, while simultaneously making demethylation reactions more favorable [27, 48, 49]. As a result, lignin degradation becomes less predictable due to the formation of undesired by‐products, ultimately yielding a faster yet less selective oxidation and a broader distribution of products.

Temperature is another significant factor that influences the kinetics and efficiency of lignin ozonolysis. Temperature affects the half‐life of ozone. As is well known for most chemical reactions, higher temperature increases the reaction rate, as predicted by Arrhenius [30]. Measured in neutral pH, and by increasing the temperature from 15°C to 35°C, the half‐life of ozone is reduced from 30 min to 8 min [50]. Practically, this means that the degradation of ozone is faster at higher temperatures; therefore, the operation window where ozone is active gets shorter. When ozone is decomposed, the only active radical species that drive the degradation of lignin are the ones shown in the scheme above. Within the ozonolysis context, accelerated degradation rates improve the overall efficiency of the lignin degradation by enhancing the diffusion rate of radicals and, simultaneously, improving the solubility of lignin in the reaction medium. However, due to the uncontrolled formation of radical species, it may also lead to excessive oxidation of lignin‐derived phenols to carboxylic acids or the complete breakdown of aromatic rings into CO_2_ and small volatile acids, hence diminishing the selectivity of oxidation products.[49, 51] Although most studies are performed at ambient temperatures, the temperature window that has been investigated without the use of a catalyst is between 0°C and 70°C[49, 51, 52, 53].

Choosing the right solvent is essential in the ozonolysis process, as it affects the solubility of lignin and ozone and the reactions between the two (Figure 3). When ozonolysis is performed between pH 2 and 6 in aqueous media, water acts as both a solvent and a reactant, promoting hydrolysis of lignin into more water‐soluble fragments [7, 37]. However, these fragments could alter the overall yield or lead to the formation of unwanted by‐products [54]. Also, water molecules can stabilize intermediate ozonide compounds through hydrogen bonding, enhancing the efficiency of the reaction. In contrast, organic solvents such as acetone, ethanol, or methanol are often chosen over aqueous alternatives to improve the selectivity of ozonolysis, as they may favor the formation of specific oxidation products by modulating the solvent's interaction with ozone or lignin [5, 41, 55]. However, polar solvents such as the ones mentioned above are less favorable in dissolving ozone than nonpolar solvents [56]. Since ozonolysis is governed by the transfer of ozone from the gas phase into the solvent, poor ozone dissolution limits the effective ozone concentration and slows the reaction rate, thus affecting the efficiency of the lignin ozonolysis process. Moreover, the absence of water reduces the risk of hydrolysis, allowing for a more controlled oxidative cleavage of lignin without excessive degradation.

### 3.4 Comparison of Ozonolysis With Other Oxidative Methods

#### 3.4.1 Oxygen‐Based Methods

Oxygen delignification is widely used in the pulp and paper industry to remove residual lignin from kraft pulp after cooking and before bleaching [57, 58]. In this process, dioxygen (O_2_) is used as the primary oxidant, typically in an alkaline environment, at elevated temperatures (80°C–120°C) and high pressures (≈0.6 MPa). Under these conditions, O_2_ forms mainly superoxides and hydroperoxides and breaks down the lignin into water‐soluble products, therefore, decreasing the need for chlorine‐based chemicals in subsequent bleaching stages. Additionally, compared to other oxidative processes, within the context of pulping, O_2_ delignification limits the degradation of cellulose, which is a significant advantage for the quality of the produced paper products [59]. For kraft pulp, oxygen delignification is more reactive at the beginning and less at the end of the process [60]. This is mainly because O_2_ shows higher reactivity towards phenolic moieties, and it is hindered by the presence of condensed structures.

Hydrogen peroxide (H_2_O_2_) is another widely used oxidizing agent in the pulp and paper industry [61, 62]. Under alkaline conditions, H_2_O_2_ generates reactive species such as HO_2_^‐^ and hydroxyl radicals, which cleave ether linkages and aromatic rings in lignin. Alkaline peroxide bleaching (pH > 10, 60°C–90°C) is essential in both mechanical and chemical pulping, because it is known to preserve the strength of cellulose [61, 63]. In contrast, acidic hydrogen peroxide bleaching produces peroxycarboxylic intermediates.

In addition to their industrial importance in pulp bleaching, O_2_ and H_2_O_2_ oxidation have been explored for direct lignin modification and valorization. Oxygen oxidation has been shown to contribute to oxidative depolymerization of lignin, leading to decreases in molecular weight and the release of water‐soluble oxidative products, indicating its potential for lignin functionalization beyond delignification [64]. On the other hand, H_2_O_2_ oxidation has been used to generate carboxyl‐rich lignin fragments with reduced molecular weight, which can be isolated and used as functional oligomers [65]. Additionally, oxidized technical lignins produced via H_2_O_2_ have been shown to exhibit useful emulsion stabilization properties, illustrating application‐driven lignin modification [66].

Peroxyacids (e.g., peracetic acid and performic acid) are strong organic oxidizing agents used for lignin removal and pulp bleaching in TCF treatments [58, 67]. They promote electrophilic attack on aromatic rings and oxidative cleavage of lignin's structure, converting lignin into low molecular weight acids and aldehydes. Peroxyacids operate efficiently at moderate temperatures (40°C–70°C) and slightly acidic pH (pH 3–5), achieving high delignification with minimal cellulose degradation. Compared to metal‐catalyzed and peroxide‐based radical oxidation, peroxyacids act as electrophilic oxidants that preferentially react with less‐condensed, electron‐rich lignins (e.g., organosolv and lignosulfonates), promoting the aromatic ring opening and the formation of more defined low molecular weight carboxylic acids. Although enzymatic oxidation may induce polymerization as mentioned above, peroxyacids avoid radical repolymerization, thus providing higher selectivity and fewer recombination by‐products. The aforementioned advantages and disadvantages of each catalytic method are summarized in Table 1 in comparison with ozonolysis.

**TABLE 1:** Comparison of lignin oxidation methods.

Oxidation method	Pros	Cons
**O** **_2_** **delignification**	‐ Reduces use of chlorine bleaching agents ‐ Low impact on cellulose	‐ Lower selectivity ‐ Limited efficiency due to lignin accessibility ‐ Elevated temperature and pressure
**H**_**2**_ **O**_**2**_ **oxidation**	‐ Mild conditions ‐ Benign by‐products ‐ Suitable for mechanical and chemical pulps	‐ Requires stabilizers ‐ Less effective for highly condensed lignin
**Peroxyacids**	‐ High selectivity ‐ Mild conditions	‐ High chemical cost ‐ Safety concerns due to instability ‐ Unwanted by‐products
**Metal‐catalyzed**	‐ Fast reaction rates (ranging from seconds to hours) ‐ Broad applicability ‐ Suitable for large‐scale processes	‐ Lower selectivity ‐ Toxicity concerns ‐ Expensive catalysts and recovery issues
**Enzymatic**	‐ High selectivity for certain lignin bonds ‐ Mild reaction conditions ‐ Biodegradable by‐products	‐ Slow reaction rates (ranging from minutes to days) ‐ Limited substrate range ‐ Enzyme instability
**Ozonolysis**	‐ Rapid and efficient (ranging from minutes to hours) ‐ No need for catalysts or enzymes ‐ High oxidation power	‐ Challenges in controlling selectivity ‐ Formation of unwanted by‐products ‐ Ozone handling difficulties

#### 3.4.2 Metal‐Catalyzed Oxidation

Metal‐catalyzed oxidation is a chemical process that utilizes metal‐based catalysts, such as iron, copper, manganese, and others, to facilitate the oxidation of compounds [68, 69]. In this process, the metal catalyst promotes the oxidative cleavage of ether bonds or aromatic rings [68]. The metal catalyst withdraws electrons from lignin, causing it to degrade radically into smaller fragments. Metal‐catalyzed oxidation can be conducted at varying temperatures and pressures, which are dependent on the type of catalyst used and the desired degradation products. For example, iron‐based catalysts, such as iron(III) chloride (FeCl_3_) and iron(III) nitrate (Fe(NO_3_)_3_), have been employed at temperatures ranging from 80°C to 250°C in organic solvents for the oxidation of lignin and the production of aromatic structures [70, 71]. Copper catalysts, such as copper(I) chloride (CuCl), copper(II) oxide (CuO), and copper(II) sulfate (CuSO4), have been used in both organic solvents and basic aqueous solvent mixtures at temperatures ranging from 30°C to 210°C, to produce aromatic compounds, such as vanillin and syringaldehyde [72, 73, 74, 75]. A significant factor influencing the efficiency of metal‐catalyzed oxidation is the structural diversity of lignin. For instance, kraft lignin is extensively condensed, which makes it less reactive toward oxidative cleavage compared to lignosulfonates or organosolv lignin, which possess higher proportions of *β*‐O‐4’ linkages and fewer condensed structures. One of the main advantages of metal‐catalyzed oxidation is its broad applicability. They can often degrade a wider variety of lignin structures, such as *β*‐O‐4’, *β*‐*β*’, *β*‐5’, and *β*‐1’ [75]. Additionally, metal catalysts offer fast reaction rates, leading to quicker lignin degradation [68, 69]. The versatility and speed of metal‐catalyzed oxidation are the main reasons why this oxidation shows promise in various industries, such as biofuel production and waste management [76, 77]. Furthermore, scalability is another significant advantage. Metal‐catalyzed processes are well‐suited for industrial‐scale operations due to their efficiency and ability to process large quantities of lignin quickly, allowing for faster processing times and the capacity to handle bulk lignin feedstocks [78, 79].

However, there are notable disadvantages associated with metal‐catalyzed oxidation [80, 81]. One of the main challenges is that metal catalysts promote non‐selective oxidation, which can result in numerous undesirable degradation products. Some metal catalysts and their by‐products, such as hydrogen peroxide or acids, raise safety and environmental concerns related to their toxicity. Finally, the cost and recovery of the catalyst are significant factors to consider [82]. Metal catalysts, while effective, can be expensive to procure and maintain. Additionally, recovering or recycling the catalysts for reuse is often challenging and costly, which may limit the overall economic feasibility, especially in large‐scale setups. Mechanistically, the reactive species involved differ from those of other oxidative methods. Metal catalysts often generate hydroxyl and superoxide radicals, and in Fenton‐type oxidation (Fe^2+^ and H_2_O_2_), highly reactive hydroxyl radicals are produced directly from hydrogen peroxide [83]. These radicals are considerably more aggressive than the peroxy radicals generated in O_2_ delignification or H_2_O_2_ bleaching, leading to deeper oxidative cleavage but also poorer selectivity [84].

#### 3.4.3 Enzymatic Oxidation

Enzymatic oxidation involves oxidoreductases such as peroxidases and laccases that rely on metal co‐factors present in their active sites. These metal ions enable efficient electron transfer and oxygen activation. Lignin peroxidase uses hydrogen peroxide as a co‐substrate to break down both phenolic and non‐phenolic lignin [85, 86, 87]. The reaction mechanism involves single electron abstraction from the phenolic of the aromatic site. The metal center is iron, coordinated within the heme co‐factor at the enzyme's active site. In manganese peroxidases, the heme iron redox reactions are coupled to the reversible oxidation of Mn^2+^ to Mn^3+^, which leads to the oxidation of mainly phenolic lignin sites. By contrast, laccases rely on a multinuclear copper active site, which couples single‐electron oxidation of phenolic lignin units to the four‐electron reduction of molecular oxygen to water [85, 86, 87, 88]. These enzymes facilitate the breakdown of lignin's structure under mild conditions, avoiding the harsh environments required by traditional chemical methods. For instance, the bacterial laccase LacZ1, with optimal activity at neutral pH, has been shown to depolymerize lignin by cleaving *β*‐O‐4’, *β*‐5’, and *β*‐*β*’ linkages and producing 4‐hydroxybenzoic acid as a degradation product [89]. Also, it was shown that a peroxidase from *Phanerochaete chrysosporium*, operating under acidic conditions and temperatures between 25°C and 45°C, can cleave *β*‐1’ and *β*‐O‐4’ linkages on lignin, producing compounds like benzaldehyde [90].

One of the key advantages of enzymatic lignin oxidation is its high selectivity. Enzymes exhibit bond‐specific reactivity toward lignin that depends on their redox potential and oxidation mechanism. Notably, laccases are selective to phenolic lignin moieties [88]. Additionally, the by‐products of enzymatic oxidation are generally biodegradable and non‐toxic, thereby reducing environmental impact [91]. However, in contrast to chemical oxidation, enzymatic systems frequently generate phenoxy radicals rather than small oxidative fragments. These radicals can undergo radical coupling reactions, which often lead to the repolymerization of lignin and, thus, the increase of molecular weight [92].

Despite their advantages, enzymatic oxidation methods present several limitations that are strongly dependent on lignin structure. Reaction rates are typically slower than those achieved with chemical oxidants such as ozone, limiting large‐scale applicability [85]. Most ligninolytic enzymes preferentially oxidize phenolic lignin units, resulting in variable reactivity depending on lignin type and isolation method [93]. Highly crosslinked or sterically hindered lignins are therefore less efficiently degraded, and chemical modifications of hydroxyl groups, such as esterification or etherification, further render lignin unreactive toward oxidoreductases by eliminating accessible phenolic sites [94]. Enzyme stability also remains a challenge, as denaturation or inactivation can occur under non‐optimal pH or temperature conditions, which is particularly relevant given the pH‐dependent redox potential of phenolic moieties [93]. Nevertheless, recent studies have demonstrated promising laccase activity in lignin degradation under alkaline conditions [95].

Compared to other oxidative methods, ozonolysis holds a unique position among oxygen‐based lignin oxidation techniques. Unlike metal‐catalyzed oxidation and enzymatic systems, ozonolysis does not require catalysts, co‐substrates, or biologically active components, allowing for rapid lignin breakdown with fewer process requirements. Compared to O_2_ delignification and H_2_O_2_‐based oxidation, ozone has a much higher oxidation potential, which enables effective breaking of aromatic rings and unsaturated side chains at lower temperatures and shorter reaction times. However, this high reactivity reduces selectivity, as radical‐based pathways can lead to over‐oxidation and unwanted by‐products. As shown in Table 1, ozonolysis presents a trade‐off between the high selectivity but slow speed of enzymatic oxidation and the faster but less selective nature of ozonolysis.

## 4 Ozonolysis for Advanced Lignin‐Based Materials and Chemicals

Recently, ozonolysis has gained increasing attention for its role in the development of advanced and functional lignin‐based materials. By cleaving the aromatic rings of lignin and oxidizing the side chains, new functional lignins with high carboxylic content, reduced molecular weight, and improved solubility and reactivity are produced. This makes ozonolysis a valuable tool for designing materials for potential high‐end applications.

Ozonated lignins with increased carboxylic content show enhanced crosslinking reactivity, enabling the creation of high‐performance polymer networks and vitrimers. For example, crosslinked vitrimer systems made with ozonated kraft lignin displayed high gel contents (≈90%), high glass transition temperatures (95°C–133°C), and up to twice the tensile strength, while remaining reprocessable through transesterification [96]. These materials also exhibited self‐healing, shape‐memory behavior, and strong adhesion (lap‐shear strength 6.5 MPa), highlighting the suitability of ozonated lignin for structural adhesives, coatings, and heat‐reversible thermosets. These findings suggest that ozonation allows property tuning through controlled functional group generation, balancing rigidity, thermal stability, and dynamic bonding.

Ozonation significantly improves lignin solubility in aqueous and polar systems by increasing the concentration of carboxylic acid groups and reducing molecular mass, enhancing surface activity [6, 55]. When further functionalized via esterification, ozonated lignin proves effective as a surfactant, reducing surface tension, providing strong emulsification comparable to commercial surfactants like Tween‐80, and offering excellent UV‐shielding and antioxidant properties [6]. These traits make ozonated lignin promising for use in cosmetics, packaging additives, stabilizers for Pickering emulsions, and eco‐friendly surface‐active materials.

Ozonated lignin also enhances compatibility and performance in polymer blend systems. Incorporating ozonated lignosulfonate into polyvinyl alcohol (PVA) membranes markedly increased hydrophilicity and water transport (2.1‐fold increase, reaching 78.6 g/m2h), while maintaining high salt rejection (≈99.9%), demonstrating advantages for desalination via pervaporation [97]. These improvements reflect better polymer–filler interactions and a more open free‐volume structure due to increased carboxylation and partial depolymerization. Such results suggest potential for ozonated lignin in advanced separation membranes, biodegradable packaging, and water‐treatment technologies.

Apart from macromolecular systems, ozonation enables reactions with colloidal particles, effectively modifying the lignin morphology and surface chemistry. Ozonolysis of lignin nanoparticles (LNPs) and crosslinked LNPs (HLNPs) resulted in reduced aromatic content, increased carboxylation, and particle fragmentation, while maintaining partial structure in more crosslinked particles [7]. Similarly, ozonation of technical lignins in ethanol led to extensive solubilization (71 wt%–87 wt%), formation of (di)carboxylic acids and esters, and improved reactivity due to lower molecular weight and increased aliphatic content [55]. These structural changes open opportunities for lignin‐based nanomaterials in drug delivery, catalysis, coatings, and responsive colloidal systems. As shown in the summarized cases, ozonolysis offers several benefits, including enhanced solubility through increased carboxylic acid groups, the ability to tailor mechanical, emulsifying, and membrane‐transport properties, and applicability to various technical lignins and nanoparticle formations.

**FIGURE 4:**
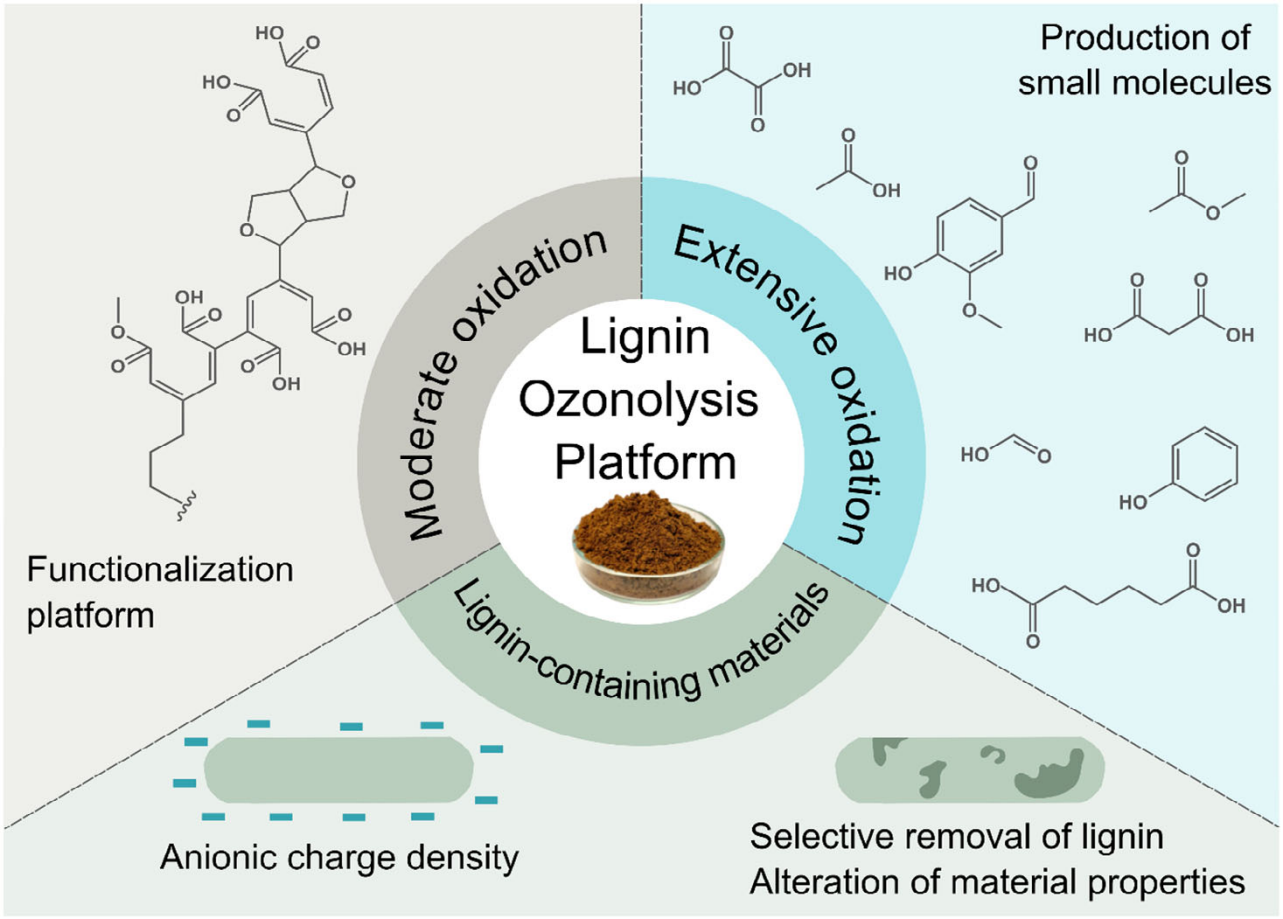
A schematic guide of the different products obtained through ozonolysis of lignin, ranging from small molecules to the modification of lignin‐containing materials. It must be noted that the moderate oxidation can produce lignin that is either soluble or colloidal.

The aforementioned studies demonstrated the potential of ozonolysis to transform lignin into a functional bio‐based alternative to synthetic, fossil‐based materials by increasing its reactivity and enhancing properties. A guide focused on the different products obtained through the ozonolysis of lignin is shown schematically in Figure 4. Overall, the extent of ozonolysis required is strongly dependent on the targeted end‐use. For applications where retaining a polymeric lignin backbone is important, such as adhesives, polymer networks, or nanoparticle systems, moderate ozonation is preferable, as it introduces oxygen groups while preserving sufficient molecular weight and structural integrity. In contrast, applications that benefit from low molecular weight oxidation products, such as surfactants, fine chemicals, or platform molecules, may require more extensive ozonolysis to fully depolymerize lignin into smaller, highly functionalized fragments. In this latter avenue, there are many untapped possibilities for upgrading the mixtures or organic acids, for instance, by funneling approaches [98] or by robust biotechnological processes [99]. Furthermore, ozonation can serve as a selective modification tool for lignin within composite materials: controlled oxidation can enhance interfacial compatibility and impart new functional properties, while more aggressive treatment may enable complete removal of lignin from the matrix. Thus, tailoring ozonolysis intensity enables a wide range of material outcomes driven by the targeted application requirements.

## 5 Challenges and Opportunities

### 5.1 Techno‐Economic Challenges and Industrial Scale Feasibility of Ozonolysis

Ozonolysis holds significant potential as an efficient method for lignin valorization. However, several technical barriers must be overcome to enhance its scalability and commercial viability. One of the main challenges in ozonolysis is achieving selective oxidation of lignin while avoiding excessive degradation. Additionally, fine‐tuning multiple reaction variables, such as ozone dosage, reaction time, temperature, and solvent choice, makes it difficult to establish optimal conditions for the process. Lignin's complex structure, variable molecular weight, and diverse functional groups make optimizing ozonolysis particularly challenging. Moreover, scaling up the ozonolysis process presents several logistical and economic challenges. Two major areas of concern are ozone generation and the need for continuous processing.

The production of ozone for ozonolysis on an industrial scale requires significant energy input, when using ozone generators working on high‐voltage electrical discharge or ultraviolet light to convert oxygen (O_2_) into ozone (O_3_), leading to notable cost implications [97, 100, 101]. Industrial ozone generators typically consume between 10 and 40 kWh of electricity to produce 1 kg of ozone, depending on efficiency and operating conditions [102]. To contextualize it, producing 1 kg of ozone can consume roughly the same amount of electricity needed to power an average U.S. household for a day (which uses around 29 kWh per day) or to run a refrigerator for a week [103]. Assuming a factory needs to process 1000 tonnes of lignin annually to be profitable, this translates to ≈150 kg of lignin per hour. The amount of ozone required depends on the intensity of ozonolysis and the possibility of recycling any unreacted ozone. For mild ozonolysis, less than 1 kg of ozone per kg of lignin is needed, whereas for extensive ozonolysis, up to 2 kg/kg may be required. This means the factory would need between 150 and 300 kg of ozone per hour, respectively. In energy terms, generating that much ozone would consume roughly 1–6 MWh, which is equivalent to the electricity needed to charge 100–150 electric vehicles [104]. It must be noted that the aforementioned ozone consumption values found are related to small‐scale ozonolysis [7, 37, 96], and such processes should be optimized for industrial operation.

**FIGURE 5:**
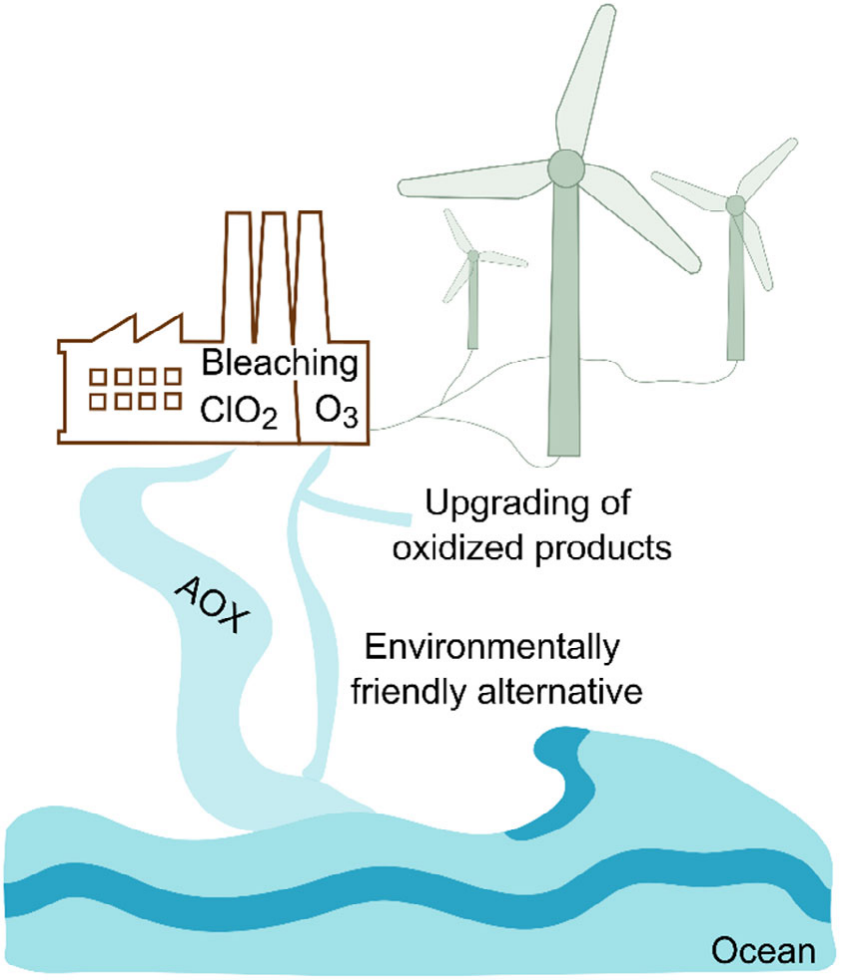
Schematic representation of the environmental benefits of ozonolysis.

In agreement with the above, recent life cycle assessment (LCA) studies have highlighted that the bottleneck of ozonolysis arises from substantial electricity demand associated with ozone generation. Similar to full‐scale ozonation used in wastewater treatments [105], in comparative LCA studies of advanced oxidation processes applied to kraft pulp bleaching, the environmental burdens of ozonation are dominated by electricity consumption required for ozone generation, which is responsible for 70%–80% of total impact [106, 107, 108]. Nonetheless, energy costs of ozonolysis must be carefully weighed against the environmental gains and potential added economic value of the lignin‐derived products, such as biofuels, bioplastics, or specialty chemicals, to ensure the feasibility of ozonolysis as a scalable lignin valorization technology (Figure 5). From the perspective of the authors, ozonolysis may find use preferentially in systems where lignin is moderately oxidized, while avoiding extensive degradation since other oxidative processes exist for producing small chemicals from lignin [109]. The benefit of ozonation is its ability to function under slightly acidic pH conditions, which may be beneficial if the material components are sensitive to acidic or basic pH. Importantly, the scalability challenges of ozonolysis may be partially mitigated by the fact that commercial bleaching processes already include ozone sequence(s) in numerous pulp mills worldwide, providing an industrial foundation for ozone handling, generation, and safety infrastructure [15, 16]. Existing ozone generators and reactors could be adapted for lignin upgrading, significantly reducing capital investment compared to completely new installations.

From an economic perspective, ozonolysis must overcome challenges in operating costs and product pricing to compete with conventional oxidation processes. Currently, fossil‐derived products are cheaper to produce due to an already established production process, tailor‐made large‐scale infrastructure, and subsidized energy costs. For example, producing one ton of bio‐based plastic from lignin could cost as much as 40% more than its fossil‐based counterpart [110]. Therefore, to make ozonolysis commercially viable, it must be competitive in terms of raw material costs, energy consumption, and operational efficiency. Government incentives such as carbon credits, tax breaks, or subsidies for renewable energy adoption could help offset the cost gap between ozonolysis and conventional processes. For example, the European Union offers subsidies for bio‐based products, which could help make ozonolysis a more competitive technology in the future.

### 5.2 Environmental Considerations

Ozonolysis offers significant environmental advantages, particularly when integrated into TCF bleaching processes, as it virtually eliminates the formation of chlorinated by‐products such as AOX in effluents. This feature reduces the release of persistent and potentially harmful compounds into water, and also aligns with circular economy principles, whereby by‐products can be valorized for various uses. Scaling ozonolysis for lignin valorization poses substantial environmental challenges that must be addressed. Energy consumption and waste management are key factors in making ozonolysis a viable replacement for traditional lignin processing methods. As described above, ozone production is highly energy‐intensive, with industrial‐scale generators consuming up to 40 kWh of electricity per kilogram of ozone produced. When scaled up, the high energy demands raise concerns about the carbon footprint of the process. One way to mitigate these environmental impacts is to integrate renewable energy sources, such as solar or wind power, into the ozonolysis process [111]. For example, a typical 1 MW wind turbine can generate enough electricity to produce 50–100 kg of ozone daily (treating up to 100 kg of lignin if low ozone is needed), which, as a result, would reduce reliance on fossil fuels and lower the overall carbon footprint of the process [112]. This shift to renewable energy would also help to reduce the economic burden of high electricity costs, thus making ozonolysis more cost‐competitive over time.

Waste management is another environmental concern associated with ozonolysis [113, 114, 115]. For instance, by‐products such as phenolic compounds and dicarboxylic acids can be used in applications like biofuels, bioplastics, or pharmaceutical intermediates. Some phenolic compounds from ozonolysis are already being used in the production of eco‐friendly adhesives and biodegradable plastics [116, 117]. However, large‐scale ozonolysis must also account for safety concerns related to the accumulation of unstable peroxidic intermediates during oxidation, which can pose explosion and handling risks [56]. Continuous‐flow reactor systems have shown promise in mitigating these hazards.

Circular economy models that prioritize the reuse of by‐products present significant opportunities to reduce waste and minimize the need for disposal. When by‐products are not immediately utilized, effective waste treatment methods can transform them safely, preventing environmental contamination. Approaches such as design for biodegradation offer promising solutions to manage these materials sustainably [118]. By integrating zero‐waste strategies, ozonolysis can become a fully closed‐loop process, equivalent to waste‐to‐energy systems that convert residual materials into valuable resources like electricity [119].

### 5.3 Future Research Directions

Future research should focus on overcoming the aforementioned limitations and improving the efficiency of ozonolysis. One promising direction is the combination of ozonolysis with catalytic or enzymatic oxidation techniques [120, 121, 122]. Using metal catalysts and enzymes along with ozonolysis can enhance reaction specificity, reduce unwanted by‐products, and improve the yield of valuable products. Improving reactor design is another vital step to enhance ozone dispersion, reaction control, and scalability [123]. While ozone is typically introduced via diffusers or spargers, achieving uniform distribution is challenging. Research should focus on continuous flow reactors, such as plug‐flow, stirred‐tank, or fluidized bed reactors, to ensure homogeneous oxidation [124]. Additionally, exploring high‐efficiency ozonators and microbubble technologies could improve ozone dissolution and ensure improved contact with lignin [125]. Finally, ozonolysis holds great potential in lignin biorefinery models, supporting circular economy solutions by converting lignin into value‐added bio‐based products. Future research should investigate how ozonolysis can be integrated with processes like fermentation or anaerobic digestion to maximize lignin valorization [31]. This could involve coupling ozonolysis with biological treatments or developing multistage processes that sequentially break down lignin into valuable chemicals.

## 6 Summary and Outlook

Ozonolysis has played a pivotal role in the pulp and paper industry and is now emerging as a valuable tool in lignin valorization, targeting sustainable and functional materials. Its ability to selectively modify lignin structures enables the development of lignin derivatives for potential high‐end applications. Nonetheless, the research front related to the material functionalization and property modification is still at its early stages. Additionally, challenges surrounding the optimization of processes and industrial scaling remain. Further research is needed on optimization protocols, hybrid oxidation approaches, and new ozonolysis reactors to improve the scalability, selectivity, and sustainability of the ozonolysis approach. Such research directions are essential to making ozonolysis a competitive and reliable means of lignin processing at the industrial scale.

## Funding

This work was supported by the Stiftelsen för Strategisk Forskning (FFL21‐0006).

## Conflicts of Interest

The authors declare no conflicts of interest.
